# Synthetic antimicrobial peptide LD4-PP protects the host against *E. coli*-induced cell death

**DOI:** 10.3389/fimmu.2025.1705805

**Published:** 2025-12-03

**Authors:** Soumitra Mohanty, John Kerr White, Yundi Yin, Taj Muhammad, Isak Demirel, Adam A. Strömstedt, Sunithi Gunasekera, Natalia Ferraz, Ulf Göransson, Annelie Brauner

**Affiliations:** 1 Department of Microbiology, Tumor and Cell Biology, Karolinska Institutet, Stockholm, Sweden; 2 Division of Clinical Microbiology, Karolinska University Hospital, Stockholm, Sweden; 3 Pharmacognosy, Department of Pharmaceutical Biosciences, Biomedical Centre, Uppsala University, Uppsala, Sweden; 4 School of Medical Sciences, Örebro University, Örebro, Sweden; 5 Nanotechnology and Functional Materials, Department of Materials Science and Engineering, Uppsala University, Uppsala, Sweden

**Keywords:** *E. coli*, synthetic antimicrobial peptide, immune response, urinary tract infection, innate immunity

## Abstract

With antibiotic resistance being a major global concern, there is a huge need of new treatment options to fight bacterial infections. In this study, we highlight the antibacterial and host-protective roles of a novel synthetic antimicrobial peptide in uropathogenic *Escherichia coli*–infected uroepithelial cells. This peptide, designed from a fragment of human cathelicidin LL-37 and named LD4-PP, was found to be highly potent against clinical isolates of *E. coli* as well as ESBL-producing and multi-drug resistant *E. coli.* Additionally, LD4-PP inhibited the formation of new biofilm, damaging both the bacterial surface and the bacterial genome. LD4-PP also modulated the host cell lipid vacuole, caveolin-1, and Rho GTPase B affecting bacterial survival. Furthermore, LD4-PP exerts immunomodulatory effects by modulating free radical formation, expression of antioxidants, and inflammasome-mediated cell death. Pronounced uroepithelial cell death was observed after *E. coli* infection which was significantly inhibited by LD4-PP without affecting the cellular toxicity. Overall, the peptide LD4-PP is shown to be a strong candidate for future clinical applications, particularly to prevent and treat urinary tract infections.

## Introduction

The emergence of antimicrobial resistance against commonly used antibiotics has resulted in the growing incidences of difficult-to-treat infectious diseases. Therefore, antimicrobial resistance is a major threat and a challenge for the treatment of patients. The risk of treatment failure and increase in complications poses an economic burden and demands for an effective alternative treatment approach (1). Interestingly, antimicrobial peptides (AMPs), which are endogenously expressed and amenable to chemical synthesis, have therapeutic values against these drug-resistant strains (2, 3).

Urinary tract infections (UTIs) are often caused by uropathogenic *Escherichia coli* where the infection is frequently self-limiting. In contrast, extended spectrum beta-lactamase (ESBL) or multi-drug resistant (MDR) isolates and infections such as acute pyelonephritis are more difficult to treat, increasing the risk of bacterial spread to the bloodstream with life-threatening consequences. AMPs are a crucial part of the innate host defense system. Most AMPs are cationic with strong bactericidal effects. Importantly, resistance development against AMPs appears slow and incomplete (4). However, toxicity to host and stability are major hurdles in pursuing AMPs for drug development and therapeutic use. Chemically synthesized AMPs, designed to have improved stability and less toxicity, could serve as an alternate treatment option. With regards to UTI, several endogenously expressed AMPs, including LL-37 (5), defensins (6), RNase7 (7), and psoriasin (8), are known to play important roles in both combating and preventing infection. We and others have also shown the antimicrobial effect of chemically synthesized peptides, in particular short sequences originating from LL-37 and cyclic dimers based on those peptides (2, 9, 10), promising potential therapeutic options against drug-resistant pathogens. Apart from antimicrobial activity, AMPs are also known to modulate the innate immune system by recruiting immune cells (11), by strengthening the epithelial barrier function (8, 12), by promoting cellular proliferation (13), angiogenesis (14), and by stimulating wound healing (15).

In the current study, we highlight the efficacy of the novel peptide LD4-PP in combating uropathogens and its immunomodulatory effect in uroepithelial cells. This peptide is designed from the potent core antimicrobial sequence of LL-37, the long peptide consisting of 12 residues (out of 37) known as KR-12. Furthermore, we also report the anti-biofilm and the protective effect on uroepithelial cells upon *E. coli* infection. LD4-PP exhibited immunomodulatory effects by altering cell surface receptors, formation of free radicals and antioxidants, and regulation of cytokines through the inflammasome pathway. Most importantly, LD4-PP prevented *E. coli* infection-induced host cell death without affecting the cell organelle structures.

## Materials and methods

### Synthesis of LD4-PP

**Figure 1:**
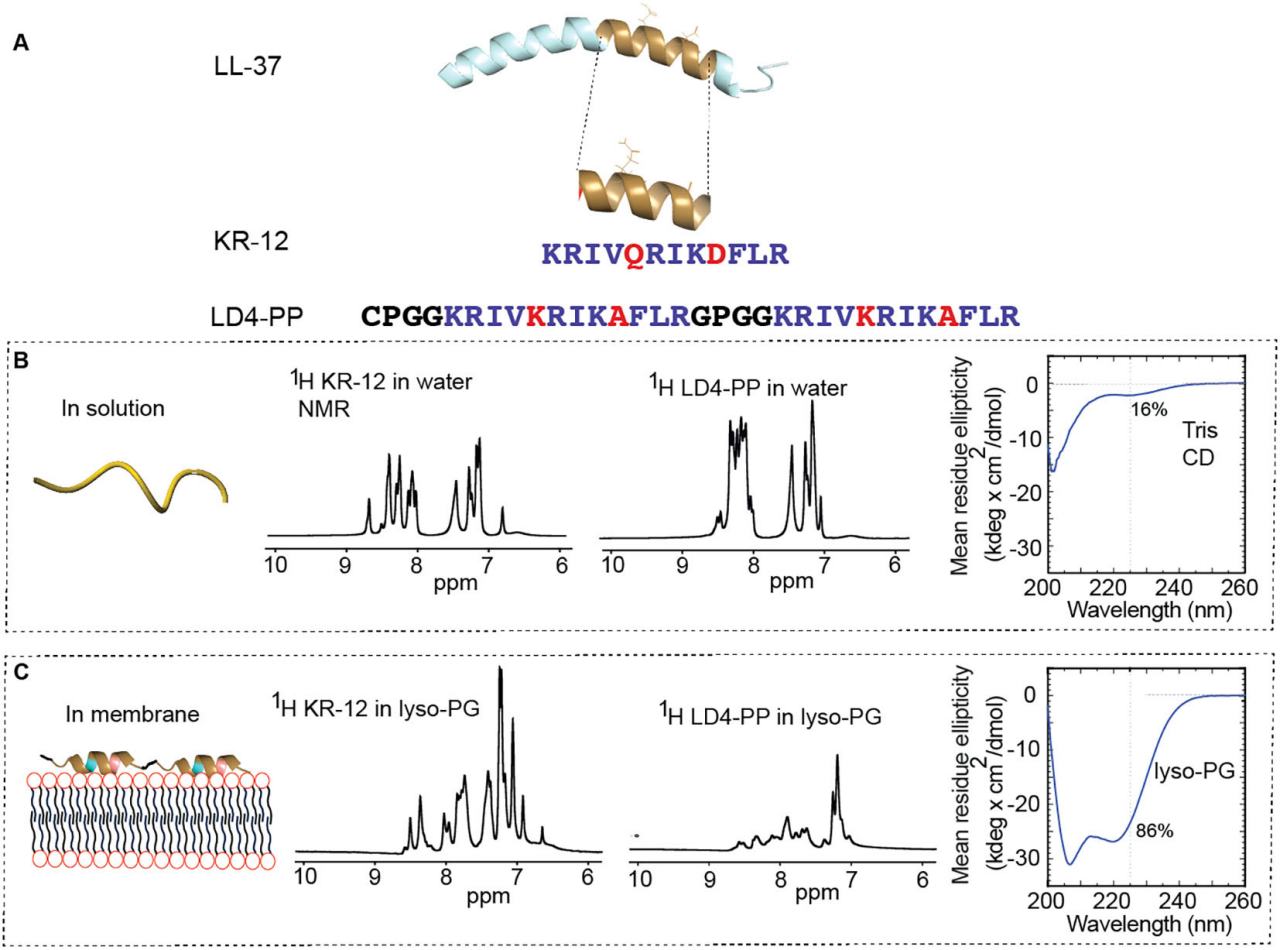
Design and structure of LD4-PP. **(A)** LD4-PP was designed by substituting the amino acid residues Q5 and D9 in the KR-12 peptide with K and A, respectively. In the final peptide, two KR-12 (Q5K, D9A) monomers form a continuous peptide backbone using four amino acid residue linkers. The C-terminus is amidated. The full sequences of KR-12 and LD4-PP are shown with the substitutions highlighted in red. When LD4-PP interacts with the lipid membrane, the structure changes from disordered in solution to mainly α-helical, as judged by membrane-mimetic models in NMR and CD. The subpanels below show 1D 1H NMR spectra of KR-12 and LD4-PP in solution and in lyso-PG (2D TOCSY and NOESY spectra are found in the **Supplementary Information**). **(B)** In solution, the chemical shift assignment was challenging, especially for LD4-PP due to significant overlap between 8 and 9 ppm, indicating unstructured conformations. **(C)** In lyso-PG, amide chemical shifts were better dispersed, with α-helical characteristics evident for KR-12. However, despite improved peak dispersion, the chemical shift assignment remained difficult for LD4-PP which was resolved by CD experiments. The mean residue ellipticity is plotted against the wavelength in CD spectra, and the relative percentage of α-helical content is determined from the signal intensity at 225 nm using poly-Lys as a reference. In Tris buffer containing micelles, the peptide adopts a predominantly α-helical profile (86% α-helical content).

LD4-PP peptide was assembled using fluorenylmethyloxycarbonyl (Fmoc)-based solid-phase peptide synthesis (SPPS) on a CEM Liberty 1 automated microwave-assisted peptide synthesizer using previously described methods (10). In brief, the C-terminal amidated peptide was synthesized on a 0.25-mmol scale using TentaGel Rink-K Amide resin (Rapp Polymere, Tubingen, Germany) and using piperidine (20% v/v in dimethylformamide) as a deprotecting agent. Large-scale purification was carried out on Claricep C-18 (20–35 μm, 100 Å) columns (Agela Technologies, Torrance, USA) using an Äkta FPLC with a flow rate of 10 mL/min. Solvents A (0% AcN, 0.05% TFA) and B (100% AcN, 0.05% TFA) were used in a linear gradient from 5% to 95% solvent B over 70 min. Final peptide purity was >95% as judged by analytical RP-HPLC-UV (215 nm), and the identity of the peptide was confirmed using LC-MS. The full sequence is CPGGKRIVKRIKDFLRGPGGKRIVQRIKDFLR, with the monoisotopic mass of 3,618.25 Da (**Figure 1A**).

### Nuclear magnetic resonance analysis

Dissolved in a mixture of H_2_O and D_2_O (9:1, v/v) at pH 5 was 1 mg of LD4-PP. NMR spectra were recorded with and without the addition of deuterated sodium dodecyl sulfate (SDS; Merck; peptide: SDS 1:40 molar ratio) or lyso-phosphatidylglycerol (lyso-PG) micelles at a concentration of 1 mM, specifically using 16:0 lyso-PG [1-palmitoyl-2-hydroxy-sn-glycero-3-phospho-(1′-rac-glycerol)] sodium salt obtained from Avanti Polar Lipids. The acquired spectra included both one-dimensional (1H) and two-dimensional (1H–1H TOCSY, 1H–1H NOESY) experiments, and the processing steps followed were consistent with previously established methods (16). The NMR analyses were performed using a Bruker Avance Neo 600 MHz spectrometer equipped with a TCI cryoprobe (CRPHe TR-1H &19F/13C/15 N5 mm-EZ).

### Circular dichroism spectrum analysis

The circular dichroism (CD) spectrum was evaluated following procedures as described previously (16). In short, the α-helical content of the peptide was assessed using a JASCO J810 spectropolarimeter (JASCO Corporation, Easton). Monitoring changes in the 200–260-nm range was conducted in a 10-mM Tris buffer at pH 7.4, with stirring, within a 1-cm quartz cuvette. Signals from the peptides, maintained at a concentration of 10 μM, were recorded both in the buffer alone and in the presence of 10 mM 16:0 lyso-phosphatidylglycerol (lyso-PG) micelles, establishing a 1:1 peptide-to-micelle ratio (equivalent to a 1:200 peptide-to-phospholipid ratio).

### Bacterial strains

Uropathogenic *E. coli* strain CFT073 *E. coli* ESBL-producing (CCUG 55197) and multidrug-resistant (MDR) *E. coli* (CCUG 62975) were used for *in vitro* experiments. A total of 10 clinical *E. coli* isolates of antibiotic-sensitive *E. coli* and ESBL-producing and MDR *E. coli* were obtained from the Department of Clinical Microbiology, Karolinska University Hospital, Sweden. The bacteria were cultured aerobically overnight on blood agar plates at 37°C, resuspended in 1× phosphate-buffered saline (PBS), measured spectrophotometrically, and confirmed by viable count.

### Minimum inhibitory concentration assay

The minimum inhibitory concentration (MIC) of LD4-PP was evaluated against the type strain and the 10 clinical bacterial isolates using a two-step micro-dilution assay (2, 17). The concentrations of LD4-PP were prepared double strength with twofold serial dilutions ranging from 10 to 0.15 µM in 50 µL of 10 mM Tris buffer, pH 7.0, in a U-bottom 96-well plate (Corning). The bacteria were grown to mid-log phase before diluting to a final concentration of 1 × 10^6^ CFU/mL in 10 mM Tris buffer using a Densichek Plus (BioMérieux). To each well, 50 µL suspension was added and incubated at 37°C for 1 h, and the final concentrations of peptide tested were from 5 to 0.078 µM. Afterward, 5 µL of 20% (w/v) tryptic soy broth (TSB) was added and incubated at 37°C for another 16–18 h, after which the MIC was measured. The MIC was read spectrophotometrically at 595 nm. MIC was defined as the lowest concentration of LD4-PP which inhibits bacterial growth.

### Scanning electron microscopy

Scanning electron microscopy (SEM) was used to evaluate bacterial morphology after treatment with LD4-PP as described previously (2). *E. coli* CFT073 was grown to mid-log phase and thereafter diluted in 10 mM Tris buffer at a cell density of 10^8^ CFU/mL. Bacterial suspensions (100 µL) were then incubated with LD4-PP [final concentration, 7.8 µM (5× the MIC at standard cell density)] for 1 h at 37°C. After the exposure experiment, bacterial suspensions (100 µL) were deposited on Nunc™ Thermanox™ coverslips (Thermofisher Scientific) and left to adhere for 1 h. The bacterial cells were then fixated with 4% paraformaldehyde (VWR) in PBS overnight at 4°C and washed 2× with PBS and deionized water. The samples were then dehydrated with a series of ethanol concentrations (10%, 30%, 50%, 70%, 90%, and 100% (v/v)) followed by further dehydration with hexamethyldisilazane (HMDS; Sigma-Aldrich) solutions (HMDS/ethanol 1:2, 2:1, and 100% HMDS). The HMDS solution was removed, and the samples were left to air-dry overnight. Coverslips were mounted on carbon stubs and sputter-coated with a conductive thin layer of gold and palladium. Bacterial cells were imaged using a LEO 1550 SEM instrument (Zeiss) with an InLens detector at 2- to 3-kV acceleration voltage and at 2- to 3-nm working distance.

### Liposomal leakage assay

The liposome production and the liposome leakage assay were performed as previously described (18). In brief, dry lipid films of *E. coli* polar lipid extract (Avanti Polar Lipids) were formed on round-bottom flask walls and re-suspended in Tris buffer containing 100 mM 5(6)-carboxyfluorescein. Multilamellar structures and polydispersity were reduced by repeated extrusion through 100-nm polycarbonate membranes mounted in a LipoFast mini-extruder (Avanti Polar Lipids). Untrapped carboxyfluorescein was removed by separation on Sephadex PD-10 columns (Cytiva). Membrane permeability was measured by monitoring carboxyfluorescein efflux from the liposomes to the external low-concentration environment, resulting in a loss of self-quenching and increased fluorescence signal.

### Biofilm formation

To investigate if LD4-PP can prevent the formation of bacterial biofilm, the crystal violet assay was used. In brief, 50 µL of 10^6^ CFU/mL of uropathogenic *E. coli* CFT073, ESBL-producing *E. coli* (CCUG 55197), 10 strains each of biofilm-forming clinical isolates of *E. coli*, MDR *E. coli*, and ESBL-producing *E. coli* were added in 150 µL LB broth without salt, with or without 5 µM of LD4-PP added. After 2 days of incubation at 37°C, the old media was discarded, and 200 µL of fresh LB broth was added to each well as described above. After an additional day of incubation, the wells were washed 3× with sterile water. Each well was stained for 15 min with 0.3% crystal violet. Non-bound crystal violet was removed, and the wells were washed 3× with PBS. The bound crystal violet was dissolved using an ethanol/acetone solution (4:1, v/v), and the absorbance was read at 570 nm.

### Cell lines and culture conditions

Human uroepithelial cells 5637 (HTB-9, American Type Culture Collection) were cultured in RPMI 1640 (Life Technologies) supplemented with 10% fetal bovine serum (FBS; Life Technologies). The cells were cultured at 37°C and 5% CO_2_.

### XTT assay

The effect of varying concentrations of LD4-PP (25 to 0.39 µM, using twofold dilutions) on uroepithelial cells 5637 metabolic activity was determined using an XTT assay as described previously (19). No cytotoxicity was observed below 10 µM as confirmed by XTT cell viability assay (**Supplementary Figure S1**), and therefore 5 µM was selected as the LD4-PP concentration to be used in all *in vitro* cell experiments.

The effect of LD4-PP on the metabolic activity of *E. coli* was also determined using the XTT assay. In brief, 50 μL from a bacterial suspension corresponding to a 0.5 McFarland standard was added to 150 μL of LB broth with a final concentration of 1.56 μM LD4-PP (corresponding to the MIC) and kept at 37°C for 24 h. The samples were then incubated with 200 μL of 20% solution of 1 mg/mL XTT (Sigma) in LB broth for 4 h. The conversion of tetrazolium salt XTT to a colored formazan derivative was measured at 450 nm in a 96-well plate. Viability controls not treated with LD4-PP were maintained throughout the cell viability assay. Media blanks were subtracted from the test strains.

### Cell infection assays

Uroepithelial cells 5637 were seeded at ~80% confluency in 24-well plates (Costar) with a multiplicity of infection (MOI) 5 of *E. coli* CFT073, ESBL-producing *E. coli* (CCUG 55971), and multidrug-resistant (MDR) *E. coli* (CCUG 62975). Infection was induced for 2 h prior to treatment with 5 µM of LD4-PP for 2 h (2). This condition was compared to untreated control. During experiments, the media did not contain FBS. After 2 h of infection, old media and non-adherent bacteria were removed by washing the wells 3× with 500 µL of PBS and fresh media, with or without LD4-PP added. The cells were re-incubated for another 2 h at 37°C with 5% CO_2_. After 2 h (total of 4 h) of infection, the old media was removed, and the cells were washed. To collect cell-associated and intracellular bacteria, the cells were lysed with 200 µL of ice-cold 0.1% Triton X-100 in PBS and scraped thoroughly. Lysates were serially diluted in PBS and plated on blood agar plates, and a viable count was performed.

### Gene expression analysis

Uroepithelial cells 5637 were seeded in 24-well plates, and four conditions were established: cells treated with or without 5 µM of LD4-PP and with or without infection with MOI 5 of *E. coli* CFT073. The cells were infected for 2 h before LD4-PP treatment and RNA isolation. Total RNA was extracted using the E.Z.N.A Total RNA Kit (Omega Bio-Tek) according to the manufacturer’s protocol. The concentration and purity of RNA were determined using a Nanodrop UV spectrometer, and up to 0.5 μg of RNA was transcribed to cDNA using the High-Capacity cDNA Reverse Transcription Kit (Applied Biosystems). Expression of target genes was analyzed using SYBR Green reagent or TaqMan reagents in Rotor-Gene PCR cycler (Corbett Life Science) with gene-specific primers or probes (**Supplementary Table S1**). Relative expressions of target genes were presented as 2^-ΔCT^ and fold change as 2^-ΔΔCT^ compared to uninfected or non-treated control.

### Immunofluorescence of cells

Uroepithelial cells 5637 were seeded in 24-well plates with 12-mm coverslips, and four conditions were established: cells treated with or without 5 µM of LD4-PP and with or without infection with MOI 5 of *E. coli* CFT073. After the required incubation time, the cells were fixed in 4% PFA for 15 min at room temperature and permeabilized with 0.1% Triton X-100 in PBS. Thereafter, the cells were blocked for an additional 60 min with the 5% BSA. The cells were stained with specific antibodies as described earlier (8). The required dilution of both primary and relevant secondary antibodies is mentioned in **Supplementary Table S2**. Thereafter, cells were counter-stained using 2.5 μg mL^-1^ 4′,6-diamidino-2-phenylindole (DAPI, Invitrogen). Confocal images were acquired on a LSM 700 microscope (Carl Zeiss) using ×63 oil immersion objective. Images were processed for intensity quantification by ImageJ software (NIH).

### Caspase 1 activity assay

Uroepithelial cells 5637 were grown in respective media to reach 80% confluence in a 96-well plate, pre-incubated with 50 µM caspase 1 substrate Ac-YVAD-AMC (Enzo Life Sciences) for 1 h at 37°C and 5% CO_2_ prior to the *E. coli* infection. The cells were infected with *E. coli* at MOI 5 and treated with 5 µM of LD4-PP at 1 h after infection. Comparisons were made between uninfected or infected cells treated with LD4-PP. Samples were analyzed after 6 h of infection with a fluorescent plate reader (Cytation 3 BioTek) at excitation/emission settings of 340/440 nm. The substrate with only medium was used as a control to subtract the basal fluorescence later.

### IL-1β release

Uroepithelial cells 5637 were grown in RPMI-1640 media to reach 80% confluence in a 96-well plate. The cells were infected with *E. coli* at MOI 5 and treated with 5 µM of LD4-PP at 1 h after infection. After a total infection time of 6 h, an enzyme-linked immunosorbent assay (ELISA) was performed to measure IL-1β (ELISA MAX Deluxe Sets, BioLegend).

### Free radical formation assay

Uroepithelial cells 5637 were treated with 5 µM of LD4-PP and infected with *E. coli*. Supernatants were collected and mixed with equal volumes of Griess reagent (Invitrogen) based on the manufacturer’s protocol after 3 h of infection. Optical density was measured at 550 nm, and free nitrite was evaluated and normalized in untreated control cells.

For total ROS analysis, 10 µM DCFH-DA (Sigma) was added to the cells, and these were incubated at 37°C and 5% CO_2_ for another 2 h. Fluorescence intensity was measured at excitation 485 nm and emission 530 nm (Fluostar Omega). Similarly, for mitochondrial ROS analysis after 1 h of *E. coli* infection, the cells were first washed with 1× Hanks’ balanced salt solution (HBSS), and then 5 µM Mitosox (Life Technologies) was added and left for 30 mins. Live cell imaging was done using Zeiss LSM 700 to measure the mitochondrial ROS. Fluorescence intensity was quantified using ImageJ.

### Statistical analysis

All statistical tests were performed in GraphPad Prism version 5. Data were obtained from Student’s unpaired *t*-test, non-parametric test using Mann–Whitney *U*-test, paired *t*-test and non-parametric one-way ANOVA, Bonferonni’s multiple comparisons, and Dunnett’s one-way ANOVA test as appropriate. Differences with *p*-values below 0.05 were considered statistically significant.

## Results

### LD4-PP design and structural change when in contact with membrane

The sequence of LD4-PP represents a dimer of the shortest antimicrobial (KR-12) sequence of the human cathelicidin peptide LL-37 (20). It includes two linker regions inherited from a cyclic variant of the same sequence, and key residue substitutions are maintained at positions 5 and 9, augmenting antimicrobial activity. The C-terminal is amidated for stabilization toward exoproteases and to facilitate automated peptide synthesis in high yield. **Figure 1A** shows the design and amino acid sequence of LD4-PP.

NMR was first used to investigate the structure of LD4-PP in solution. 1D and 2D spectra revealed limited dispersion and broadened peaks, indicating an unstructured state in aqueous conditions. To investigate potential structural changes in environments resembling bacterial membranes, SDS–micelles were introduced. However, the complexity of the NMR spectra rendered precise assignments impossible, and the exact structure remained unknown. Then, 16:0 lyso-phosphatidylglycerol (lyso-PG) micelles were used instead of SDS. These micelles mimic the anionic composition of microbial membranes, with a diameter comparable to biological membrane thickness, and were employed at a 1:1 peptide-to-micelle ratio. Due to substantial spectral overlap and the high concentration of lyso-PG, the spectra were again challenging to assign, although amide protons were slightly better dispersed. **Figures 1B, C** show the 1H spectra, and TOCSY and NOESY spectra of LD4-PP are found as shown in **Supplementary Figure S2**.

In contrast to NMR, circular dichroism (CD) spectroscopy provided insight into the secondary structure. CD spectra of the peptide were recorded in Tris buffer with and without lyso-PG micelles. LD4-PP displayed random coil confirmations in the absence of micelles, consistent with the NMR experiments above; in the presence of micelles, the peptide is predominantly α-helical.

### LD4-PP shows potent antibacterial activity and damages the bacterial membrane

The MIC of LD4-PP was evaluated against type strains and clinical uropathogenic bacterial isolates, including both sensitive and resistant *E. coli* species. The MIC was determined to be 1.56 µM against both type and clinical strains (*n* = 2 and *n* = 10, different isolates).

**Figure 2:**
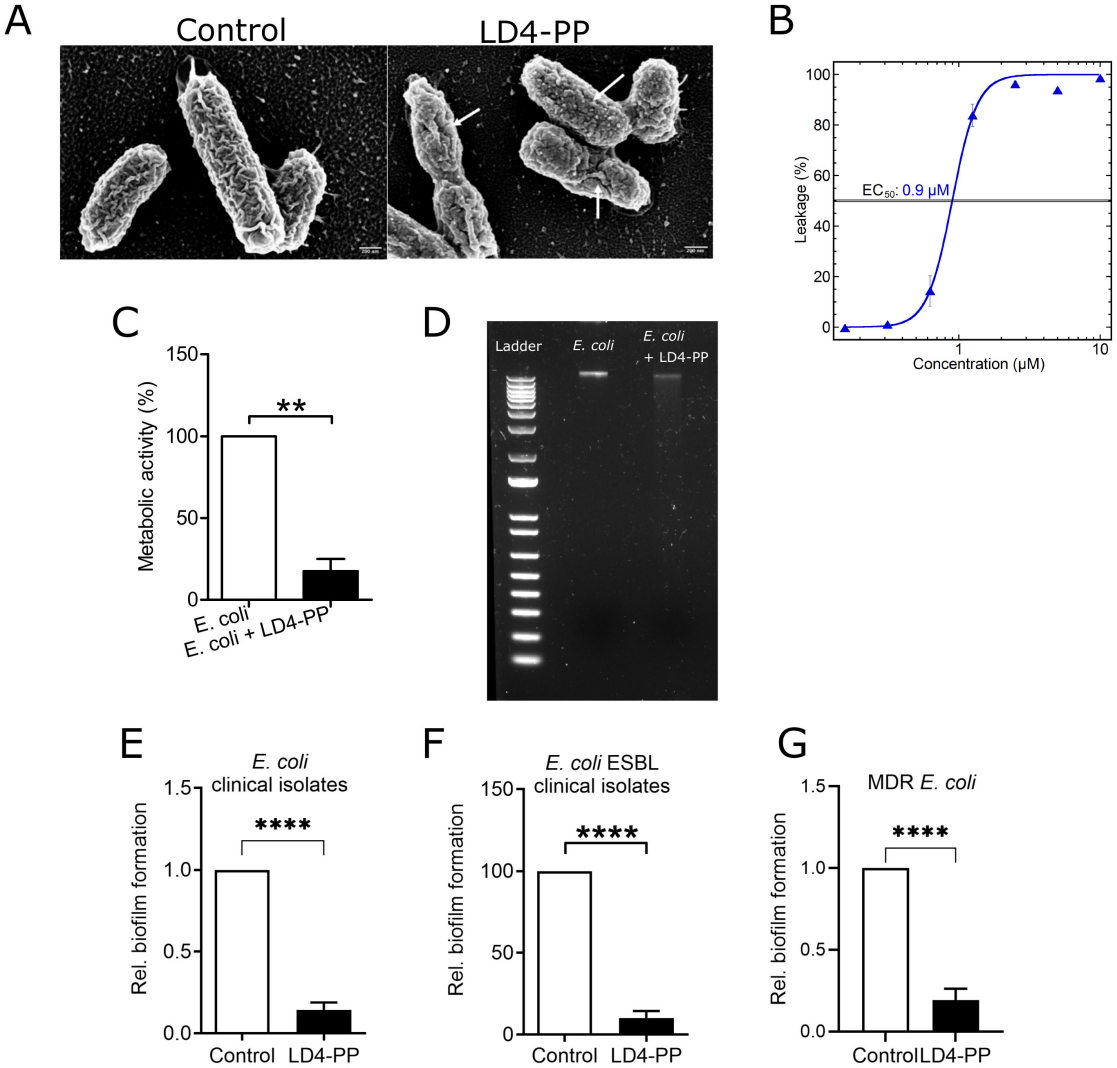
LD4-PP showed potent antimicrobial activity. **(A)** Representative scanning electron microscopy images of LD4-PP-treated and untreated *E*. *coli*. Marked increase in surface roughness, few blebs, and membrane damage (marked with arrows) were observed in LD4-PP-treated E. coli in comparison to the untreated condition (*n* = 3). **(B)** LD4-PP-induced liposome leakage was assessed on *E*. *coli* liposomes. LD4-PP induced leakage at sub-micromolar concentration with an EC_50_ of 0.9 μM. The results are the means from triplicate experiments with standard deviations. **(C)** Metabolic activity of *E*. *coli* determined using XTT assay at 24 h after LD4-PP treatment, expressed as the percentage of untreated *E*. *coli* (*n* = 3). **(D)** The genomic DNA of *E*. *coli* was isolated, and fragmentation of DNA was observed in 1.56 µM LD4-PP-treated *E*. *coli*, indicating significant genomic instability in comparison to the untreated genome (*n* = 3). The inhibition of new biofilm formation was analyzed after 72 h of incubation; 5 µM LD4-PP prevented biofilm formation by clinical strains of **(E)***E*. *coli* (*n* = 10), **(F)** ESBL-producing *E*. *coli* (*n* = 10), and **(G)** MDR *E*. *coli* (*n* = 10). Data are shown as normalized to control and are mean ± SEM. Significance levels were mentioned as **p ≤ 0.01 and ****p ≤ 0.0001.

Cationic AMPs are known to act on the negatively charged bacterial cell wall. To examine the effect of LD4-PP, the morphology of LD4-PP-treated and untreated *E. coli* CFT073 was observed under a scanning electron microscope (SEM). The LD4-PP treatment resulted in a marked increase in membrane roughness, the presence of blebs throughout the bacterial surface, and evidence of membrane damage when compared to the untreated control (**Figure 2A**). Furthermore, the direct interaction with membranes was demonstrated with a liposome leakage assay using *E. coli* phospholipids. LD4-PP induced leakage at sub-micromolar concentration with an EC_50_ of 0.9 μM, as shown in **Figure 2B**. The leakage kinetics and concentration dependence were similar in nature to that of LL-37, implicating bacterial membrane disruption as the mechanism responsible for the observed bactericidal activity.

### LD4-PP affects metabolic activity and inhibits biofilm formation

To assess the bacterial metabolic activity of *E. coli*, we performed the XTT assay upon LD4-PP treatment. The metabolic activity of LD4-PP-treated *E. coli* was reduced by almost 75% compared to untreated *E. coli* (**Figure 2C**). Furthermore, genomic DNA was isolated from control and LD4-PP-treated *E. coli*, clearly showing a smear indicating the fragmentation of genomic DNA with 1.56 µM LD4-PP 1-h treatment in *E. coli* (**Figure 2D**).

Metabolically active bacteria can form a biofilm, which protects them from external threats such as host defense peptides and antibiotics. Hence, we also tested if LD4-PP could inhibit the formation of biofilm caused by antibiotic-sensitive, ESBL-producing, and MDR-*E. coli.* Treatment with LD4-PP showed a significant reduction, nearly eradicating the production, of biofilm produced by antibiotic-sensitive, ESBL-producing, and MDR *E. coli* (**Figures 2E–G**).

### LD4-PP protects uroepithelial cells from *E. coli* infection

**Figure 3:**
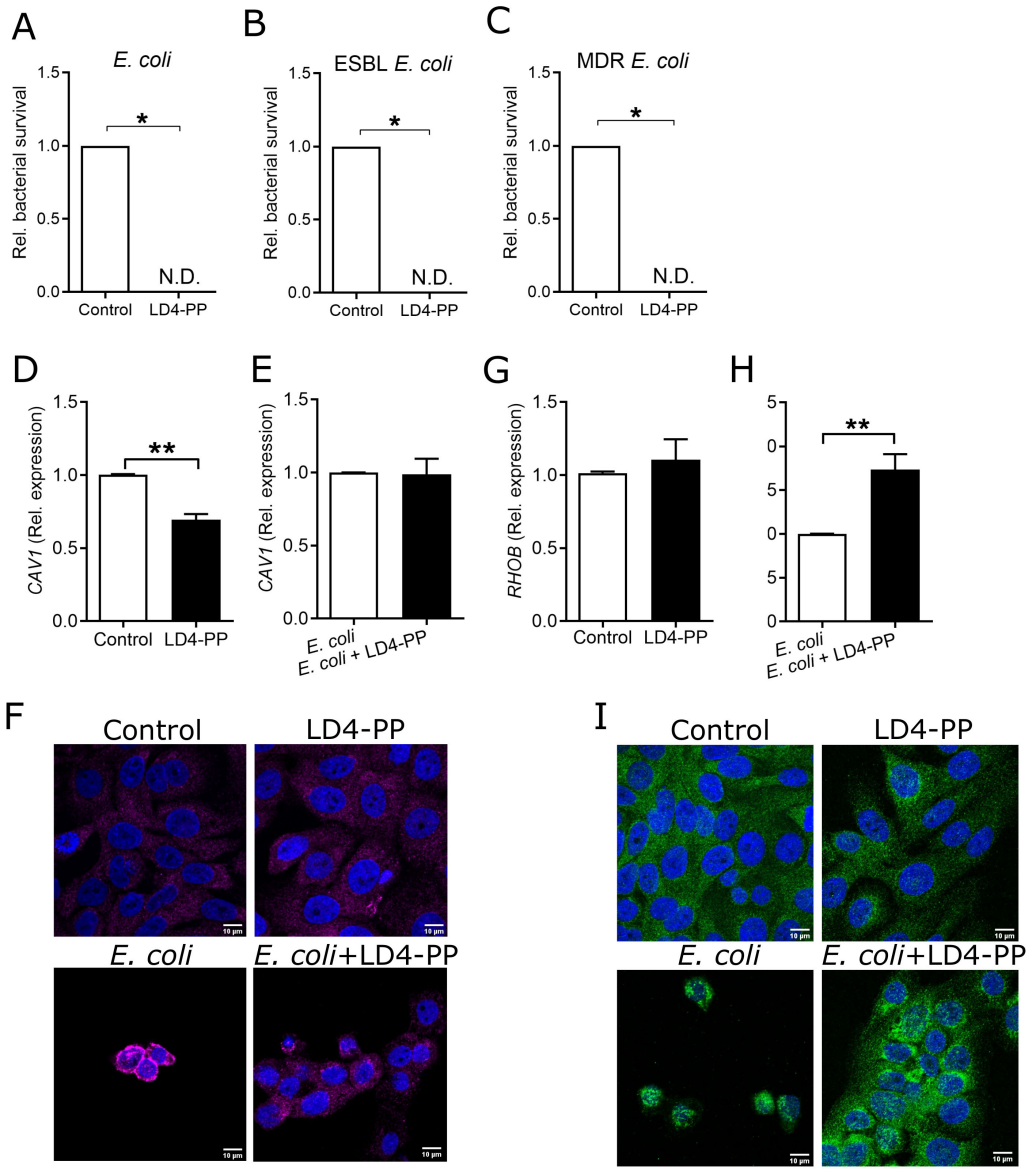
LD4-PP differentially regulates cell surface receptors. Survival of uropathogens **(A)***E*. *coli* CFT073, **(B)** ESBL-producing *E*. *coli* (CCUG 55197), and **(C)***E*. *coli* multidrug-resistant (MDR) (CCUG 62975) after infection in uroepithelial cells 5637 followed by treatment of 5 µM LD4-PP 2 h post-infection (*n* = 3). ND, not detected. Expression of *CAV1* mRNA **(D)** upon 5 µM LD4-PP treatment and **(E)** at 1 h after infection followed by 1 h of 5 µM LD4-PP treatment in human uroepithelial cells 5637 (*n* = 4). **(F)** Intracellular caveolin-1 was stained with Alexa-647 (magenta) and DAPI (blue) for LD4-PP alone at 2 h after *E*. *coli* infection or 1 h after infection followed by 1 h of LD4-PP treatment (*n* = 3). Similarly, expression of *RHOB* mRNA **(G)** upon 5 µM LD4-PP treatment and **(H)** at 1 h after infection followed by 1 h of 5 µM LD4-PP treatment in human uroepithelial cells 5637 (*n* = 4). **(I)** Intracellular RhoB was stained with Alexa-488 (green) and DAPI (blue) for LD4-PP alone at 2 h after *E*. *coli* infection or 1 h after infection followed by 1 h of LD4-PP treatment (*n* = 3). Data are shown as mean ± SEM. Significance levels were mentioned as *p ≤ 0.05 and **p ≤ 0.01.

After observing the promising direct antibacterial activity, we investigated the effect of LD4-PP in the treatment of infected uroepithelial cells. To mimic the natural situation, treatment at 2 h after infection was initiated. Interestingly, we observed a complete eradication of attached/intracellular bacteria for *E. coli* CFT073 (**Figure 3A**), ESBL-producing *E. coli* (**Figure 3B**), and MDR *E. coli* (**Figure 3C**).

### LD4-PP modulates uroepithelial cell membrane proteins

Given the results showing significantly less infection in LD4-PP-treated uroepithelial cells, we hypothesized whether this could be due to indirect effects, potentiating the direct effect, such as causing alterations in cell membrane proteins. During UTI, after bacterial attachment to uroepithelial cells, the cell surface protein caveolin 1 influences the endocytic uptake of *E. coli* by forming a flask-shaped caveolae (21). Interestingly, LD4-PP treatment on uninfected human uroepithelial cells resulted in a lower expression of *CAV1* compared to the untreated control (**Figure 3D**). In contrast, when treating cells infected with *E. coli*, we did not observe any changes in the expression of caveolin-1, neither on the mRNA (**Figure 3E**) nor protein level (**Figure 3F**) compared with the infected, non-treated controls, indicating that infection treatment lacked effect.

Endocytosis of invading *E. coli* via caveolae is also influenced by Rho proteins, which localize to caveolae and interact with caveolins (22). Therefore, we investigated the effect of LD4-PP on Rho GTPase B which is known to play an important role in intracellular bacterial survival. Surprisingly, we did not see any effect on RhoB by LD4-PP alone (**Figure 3G**), neither on the mRNA nor on the protein level (**Figure 3I**). However, in contrast to the caveolin-1 results in the infection condition, LD4-PP increased the expression of RhoB both on the mRNA (**Figure 3H**) and protein level (**Figure 3I**) in *E. coli*-infected cells.

Other cell membrane genes involved in bacterial attachment and invasion, like *ITGB1*, were only slightly increased by LD4-PP in uninfected cells, while *UPK1A* was not influenced in either uninfected or infected cells (**Supplementary Figure S3**).

### LD4-PP differentially regulates free radical formation

**Figure 4:**
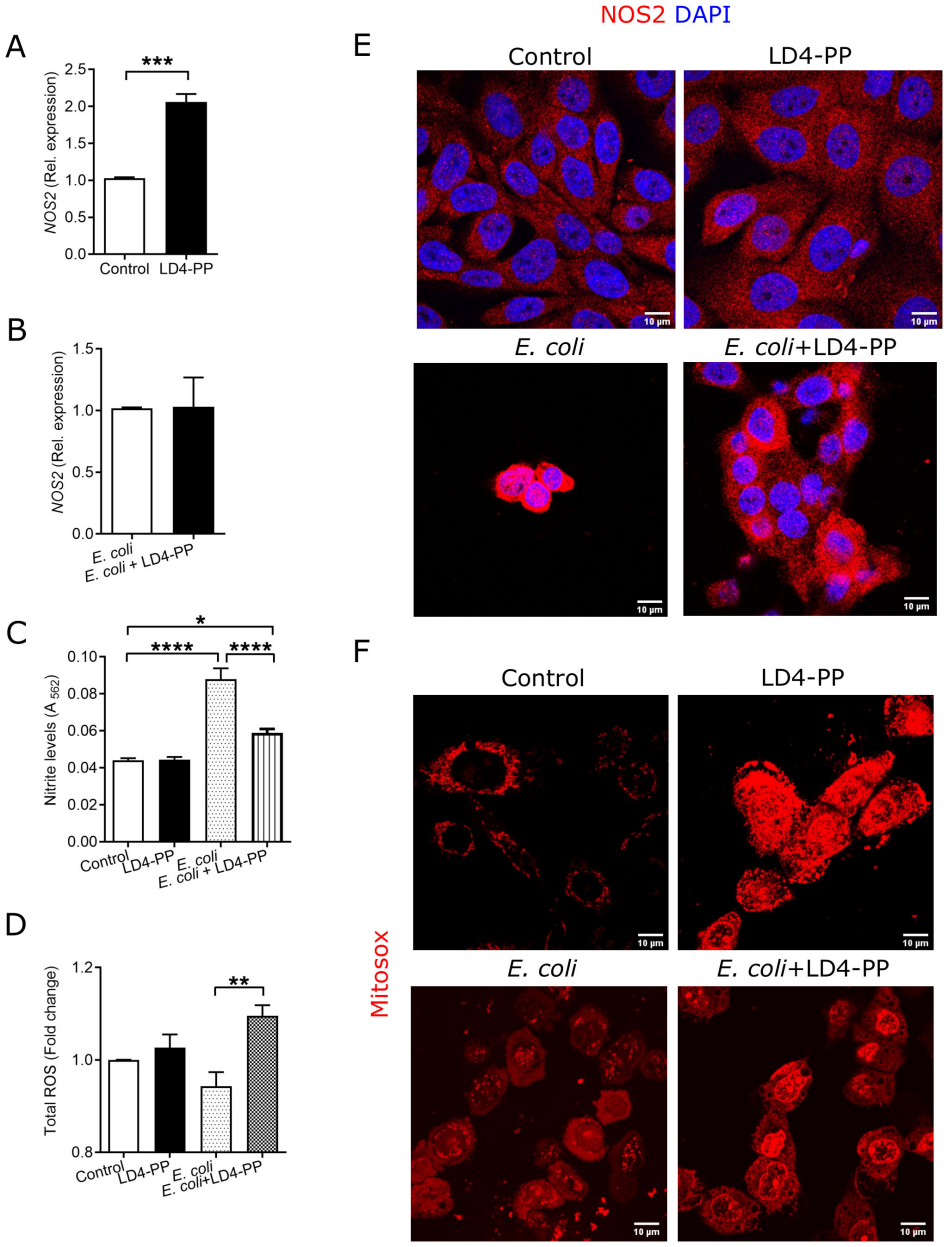
LD4-PP modulates free radical formation. Expression of *NOS2* mRNA **(A)** upon 5 µM LD4-PP treatment and **(B)** at 1 h after infection followed by 1 h of 5 µM LD4-PP treatment in human uroepithelial cells 5637 (*n* = 4). **(C)** Intracellular NOS2 was stained with Alexa-594 (red) and DAPI (blue) for LD4-PP alone at 1 h after infection followed by 1 h of LD4-PP treatment (*n* = 3). **(D)** Free NO was measured in non-treated uroepithelial cells 5637, treated with 5 µM of LD4-PP, *E coli* infected for 3 h, or at 1 h after infection treated with LD4-PP and human uroepithelial cells 5637 after treatment with 5 µM of LD4-PP (*n* = 4). **(E)** Total intracellular ROS in *E*. *coli*-infected cells after 3 h, non-infected human uroepithelial cells 5637, after treatment with 5 µM of LD4-PP, *E*. *coli*-infected cells after 2 h, followed by 1-h treatment of LD4-PP (*n* = 4). **(F)** For mitochondrial ROS, the mitochondria were stained with mitosox (red) for LD4-PP alone, at 1 h after *E*. *coli* infection or at 30 min after infection, followed by 30 min of LD4-PP treatment (*n* = 3). Data are shown as mean ± SEM. Significance levels were mentioned as **p ≤ 0.01, ***p ≤ 0.001, and ****p ≤ 0.0001.

Free radical formation is an important innate immune response upon infection. Interestingly, *NOS2* mRNA was upregulated in LD4-PP-treated uroepithelial cells when treated with LD4-PP (**Figure 4A**), but when treated after *E. coli* infection, the effect was outcompeted (**Figure 4B**). Similarly, in agreement with the mRNA levels, LD4-PP alone significantly increased the expression of NOS2 at the protein level. *E. coli* infection further increased the expression of NOS2, whereas in LD4-PP treated cells, we observed no marked change in the expression of NOS2 levels at the protein level (**Figure 4C**). However, free nitrite levels did not show any difference between LD4-PP and untreated cells, whereas *E. coli* infection resulted in increased nitrite levels (**Figure 4D**). This effect was alleviated by LD4-PP treatment of infected cells. In contrast, LD4-PP treatment after infection resulted in slightly increased levels of total intracellular ROS as compared with the control (**Figure 4E**). Similarly, LD4-PP treatment resulted in increased levels of mitochondrial ROS. In contrast, we observed no clear difference in mitochondrial ROS between *E. coli*-infected and LD4-PP-treated cells and control cells infected with *E. coli* (**Figure 4F**).

**Figure 5:**
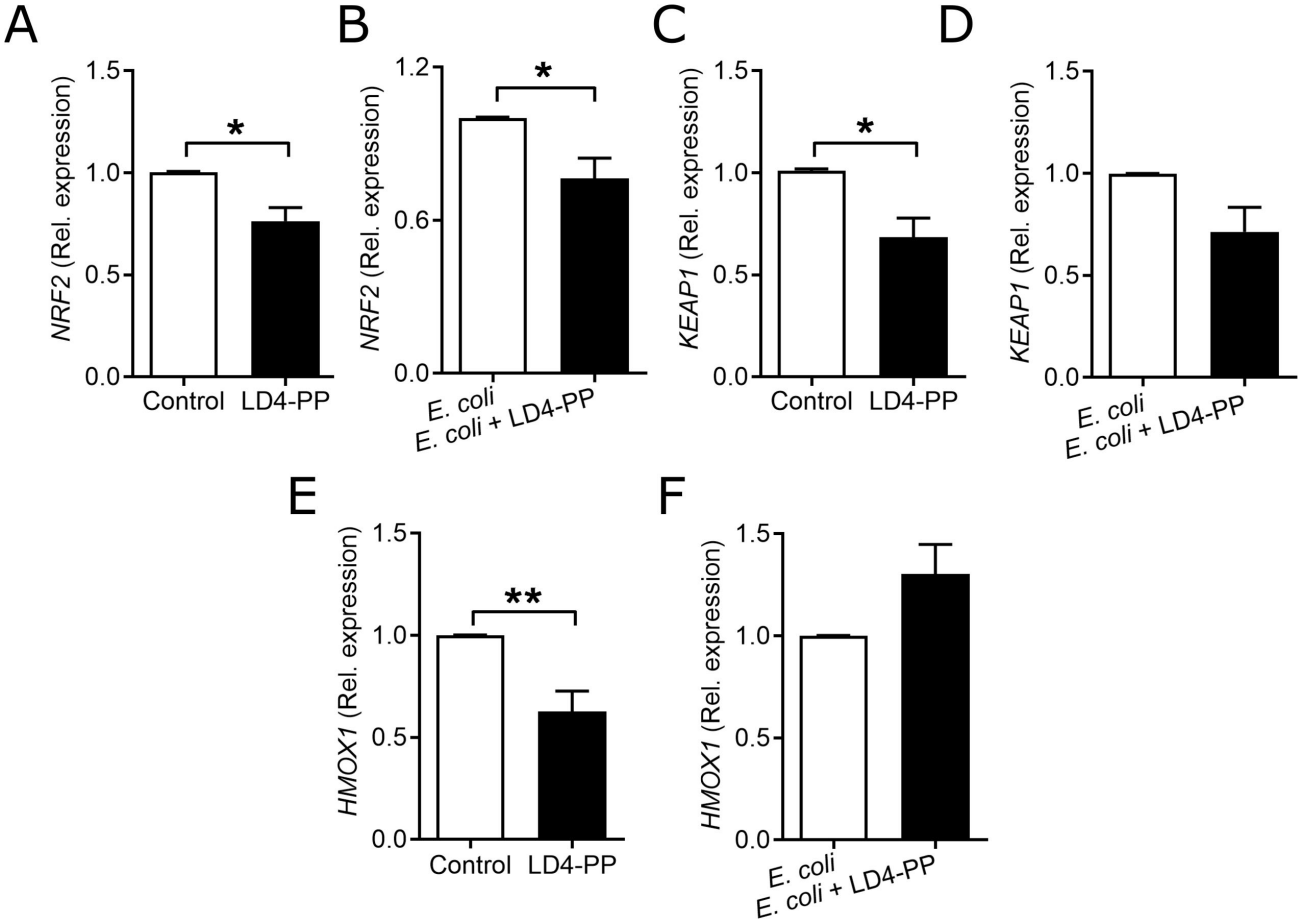
LD4-PP alters the expression of antioxidants. Expression of *NRF2* mRNA **(A)** upon 5 µM LD4-PP treatment and **(B)** at 2 h after *E*. *coli* infection followed by 5 µM LD4-PP treatment in human uroepithelial cells 5637 (*n* = 4). Expression of *KEAP1* mRNA **(C)** upon 5 µM LD4-PP treatment and **(D)** at 2 h after *E*. *coli* infection and 1-h treatment of 5 µM LD4-PP in human uroepithelial cells 5637 (*n* = 4). Expression of *HMOX1* mRNA **(E)** upon 5 µM LD4-PP treatment and **(F)** at 1 h after infection, followed by 1 h of 5 µM LD4-PP treatment in human uroepithelial cells 5637 (*n* = 4). Data are shown as mean ± SEM. Significance levels were mentioned as *p ≤ 0.05 and **p ≤ 0.01**.

Upon response to free radicals, host cells express antioxidants to limit excessive self-damage. Since an increase in the expression of free radicals in LD4-PP-treated uroepithelial cells was observed, we expected compromised levels of regulators of antioxidants, like nuclear factor erythroid 2 related factor 2 (NRF2). Interestingly, we observed a decreased expression of *NRF2* in uninfected and LD4-PP-treated cells, respectively (**Figure 5A**), and when treating infected cells with LD4-PP (**Figure 5B**). Similar observations were observed for *KEAP1* mRNA (**Figures 5C, D**). Furthermore, to support the effect of NRF2 translocation, NRF2 target genes like *HMOX1* were analyzed and found to have decreased mRNA expression in LD4-PP-treated uroepithelial cells (**Figure 5E**) but did not alter after *E. coli* infection (**Figure 5F**).

### LD4-PP-mediated inflammasome pathway and secretion of proinflammatory cytokines

**Figure 6:**
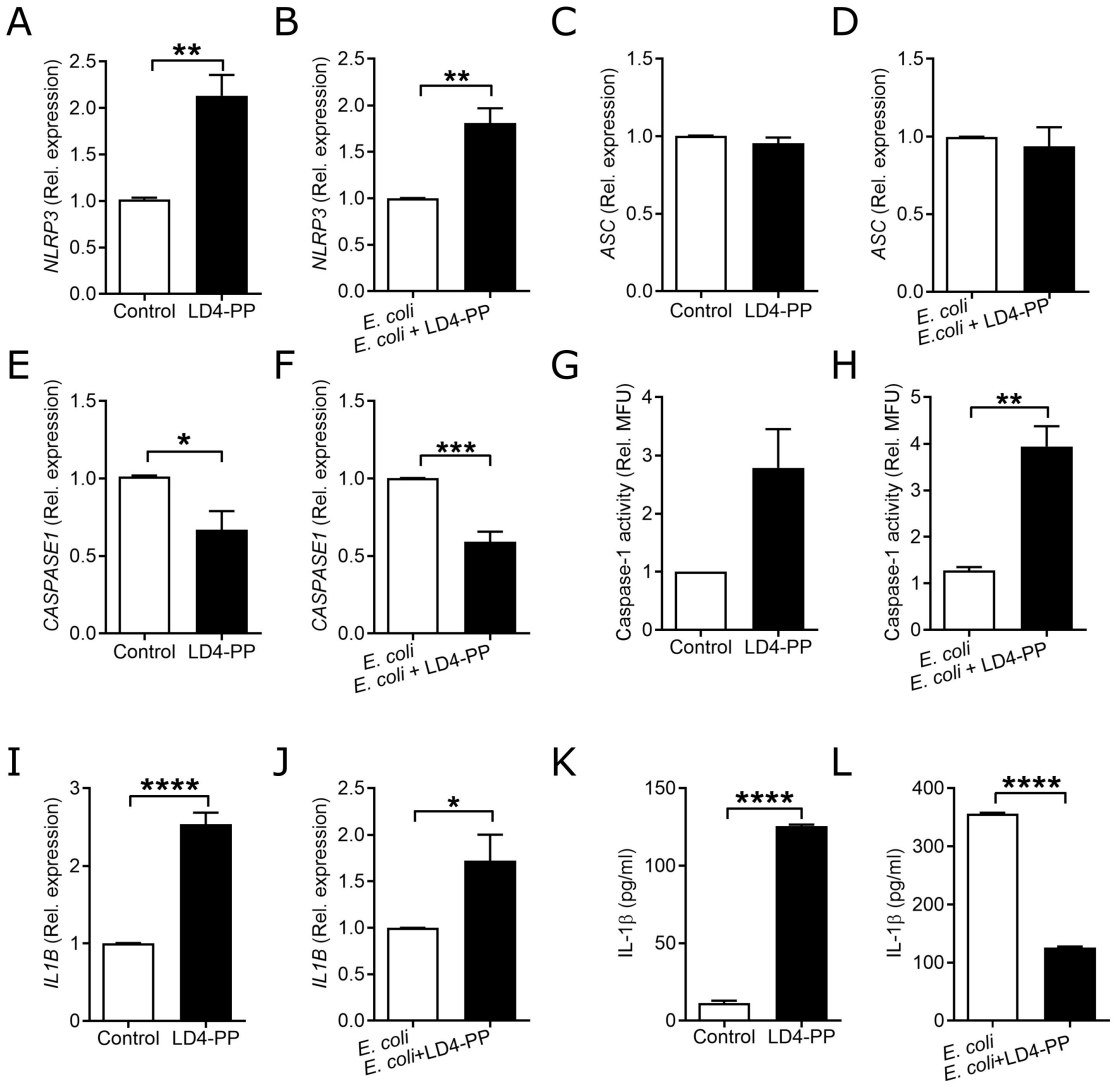
LD4-PP regulates the inflammasome pathway. Expression of *NLRP3* mRNA **(A)** upon 5 µM LD4-PP treatment and **(B)** at 2 h after *E*. *coli* infection and 5 µM LD4-PP treatment in human uroepithelial cells 5637 (*n* = 4). Expression of *ASC* mRNA **(C)** upon 5 µM LD4-PP treatment and **(D)** at 2 h after *E*. *coli* infection and 1 h treatment of 5 µM LD4-PP treatment in human uroepithelial cells 5637 (*n* = 4). Expression of *CASPASE1* mRNA **(E)** upon 5 µM LD4-PP treatment and **(F)** at 2 h after *E*. *coli* infection and 1 h treatment of 5 µM LD4-PP treatment in human uroepithelial cells 5637 (*n* = 4). Relative caspase 1 activity assay upon 6 h of *E*. *coli* infection or after only **(G)** LD4-PP treatment (*n* = 3), and **(H)** after *E*. *coli* infection (*n* = 3). Expression of *IL1B* mRNA **(I)** upon 5 µM LD4-PP treatment, **(J)** at 2 h after *E*. *coli* infection and 1 h infection, followed by 1 h of 5 µM LD4-PP treatment in human uroepithelial cells 5637 (*n* = 4). Expression of IL-1β peptide **(K)** upon 5 µM LD4-PP treatment and **(L)** after 3 h of infection followed by 3 h of 5 µM LD4-PP treatment in human uroepithelial cells 5637 (*n* = 3). Data are shown as mean ± SEM. Significance levels were mentioned as *p ≤ 0.05, **p ≤ 0.01, ***p ≤ 0.001, and ****p ≤ 0.0001.

*E. coli* infection triggers the inflammasome-mediated pathway, which, in turn, activates inflammatory responses and cell death, such as NLRP3 and Apoptosis-associated Speck-like protein containing a CARD (ASC). Therefore, we investigated the effect of LD4-PP on inflammasome markers during *E. coli* infection. LD4-PP treatment significantly increased the mRNA of *NLRP3* in uninfected cells (**Figure 6A**) and when cells were infected with *E. coli* and treated (**Figure 6B**), whereas no difference was observed in the *ASC* expression (**Figures 6C, D**) with decreased expression of *CASPASE1* (**Figures 6E, F**) when compared to the respective control cells. LD4-PP alone did not significantly increase the caspase 1 activity (**Figure 6G**). However, after 6 h of *E. coli* infection, caspase 1 activity was increased in LD4-PP-treated cells (**Figure 6H**).

The increased expression of caspase 1 activity resulted in an increased expression of *IL1B* mRNA in LD4-PP-treated (**Figure 6I**) and *E. coli*-infected uroepithelial cells (**Figure 6J**) with a similar observation for *IL6* and *CXCL8* mRNA (**Supplementary Figure S4).** However, IL-1β ELISA revealed increased levels with LD4-PP only at the uninfected state (**Figure 6K**) and with decreased levels in LD4-PP-treated *E. coli*-infected cells (**Figure 6L**). This observation could be due to the increased levels of IL-1β triggered by *E. coli* infection.

### LD4-PP protects host cells from *E. coli*-induced cell death

**Figure 7:**
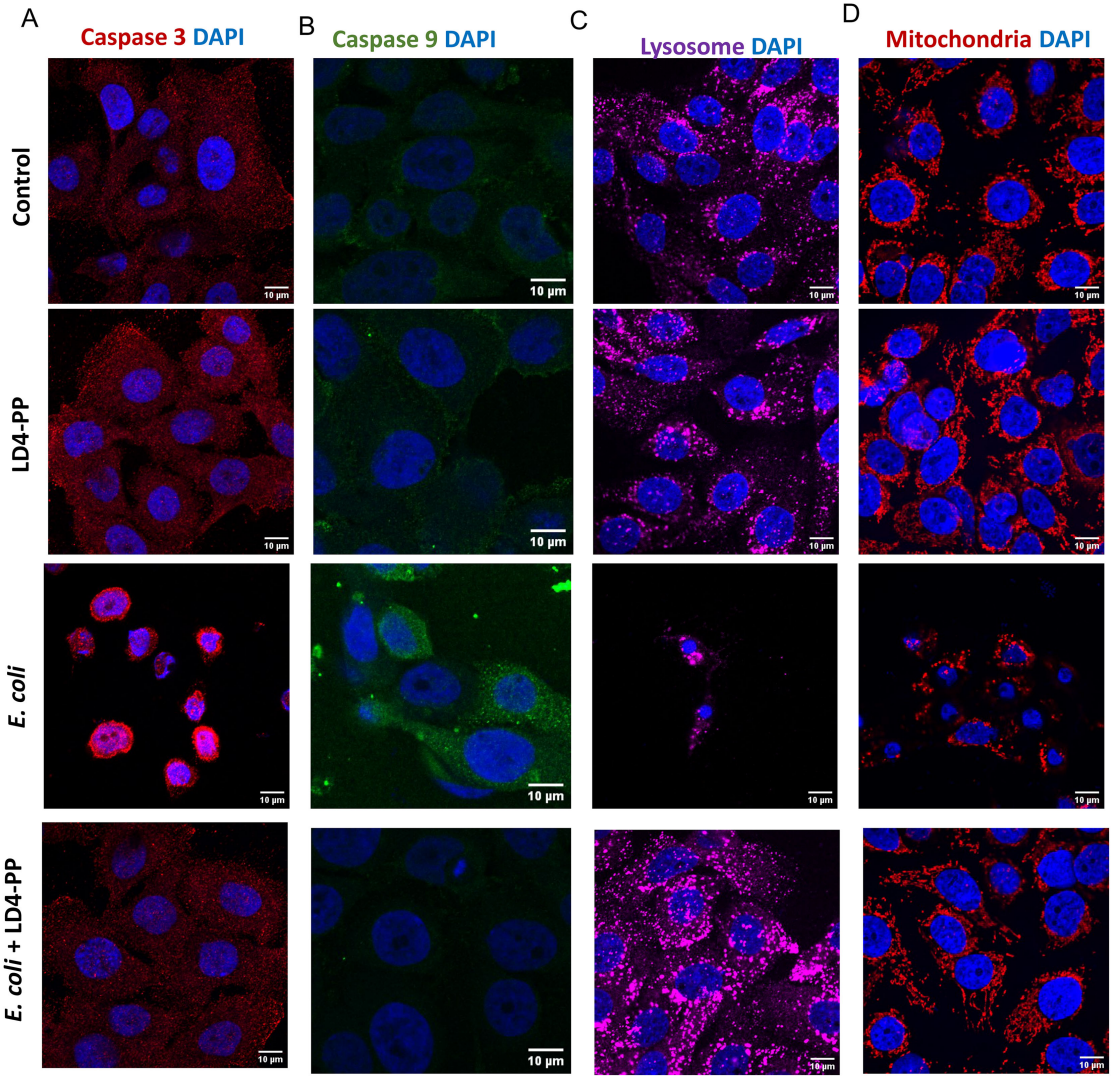
LD4-PP protects *E*. *coli*-infected uroepithelial cells with no effect on cell organelles. Expression of **(A)** Caspase 3, **(B)** Caspase 9, staining of **(C)** lysosome, and **(D)** mitochondria upon 5 µM LD4-PP treatment at 2 h after *E*. *coli* infection together with 5 µM LD4-PP and at 1 h after *E*. *coli* infection, followed by 1-h treatment of LD4-PP (*n* = 3); a representative image is presented.

In addition to their immunomodulatory influence, AMPs can be toxic to cells depending on the concentration. Although we used a non-cytotoxic concentration, we were interested in investigating a possible impact on cell death and therefore analyzed specific cell death markers. Apoptotic markers caspase 3 (**Figure 7A**) and caspase 9 (**Figure 7B**) revealed increased expression due to *E. coli* infection, whereas LD4-PP treatment nullified the effect of infection. Furthermore, the staining of relevant cell organelles, lysosome (**Figure 7C**), and mitochondria (**Figure 7D**) confirmed the toxic effect caused by infection and the rescue by treatment with LD4-PP.

## Discussion

Appropriate clinical antibiotic stewardship is made difficult with the increase in broad-range antibiotic resistance, thus calling for immediate action. We speculate that AMPs can serve as a complement or potential alternative to traditional antibiotics due to their high and broad antimicrobial activity, lower rate of resistance development, and positive host immunomodulatory effects as a future treatment regime. Presently, the intrinsic instability of natural AMPs poses a major challenge for their usage in clinical settings, but chemical modifications can significantly increase their stability (23).

The basis for LD4-PP peptide design was based on our previous work by combining two monomers, each featuring two amino acid substitutions and originating from the shortest antibacterial peptide sequence identified in LL-37 (known as KR-12) (16, 20). Significantly, each linker comprises a proline residue to introduce a kink in the backbone to potentially facilitate the folding of the peptide into two adjacent α-helices. To assess the success of the structural design, NMR spectroscopy was initially employed. However, the assignment of residues proved challenging due to overlapping signals and poor peak dispersion, suggesting a disordered peptide. The use of lyso-PG micelles did improve dispersion, indicating some elements of structure. The use of CD then revealed the secondary structure of the peptide; in buffer, LD4-PP adopts a random coil conformation, transitioning into a broadly α-helical conformation in membrane-mimicking environments. This transition is analogous to the parent peptide, KR-12.

The ability to separate internal from external components is essential for a cell to survive. Therefore, for bacteria to multiply and survive, membrane integrity is fundamental. We observed an increased number of bacterial blebbing on the LD4-PP-treated *E. coli* surface as well as significant changes in the membrane surface. Stressors, including AMPs, are known to alter the peptidoglycan of the cell wall, leading to membrane blebbing, a known determinant of integrity loss (24). Bacterial metabolic activity was also impacted by LD4-PP, which is in line with previous reports where synthetic and endogenous AMPs are known to inhibit metabolic activity by inhibiting the cell wall, nucleic acid, protein synthesis, or enzymatic activity (25). AMPs, being positively charged, are well known for making pores on the bacterial surface. The impact of entry by these small peptides can be investigated by evaluating the impact on the genetic material of the bacteria. Interestingly, we observed that LD4-PP can degrade the bacterial DNA at the MIC concentration, thereby suggesting a possible genotoxic effect, similar to the AMP clavanin A, which triggers DNA damage in *E. coli* (26).

Biofilm is a major hurdle to effective antibiotic treatment in UTIs. It is well known that multiple bacterial species reside in the mature biofilm and often complicate the antibacterial treatment. Interestingly, LD4-PP was found to be potent in inhibiting the biofilm formation of both sensitive and multidrug-resistant *E. coli* clinical strains, highlighting the broad-spectrum ability of LD4-PP.

Apart from its direct *in vitro* antibacterial activity, LD4-PP was also found to be highly potent in killing bacteria infecting human uroepithelial cells. This observation could be due to the direct antimicrobial activity of LD4-PP alone, but peptide-induced alterations in the host cell response could be a contributing factor. We observed that LD4-PP treatment alone reduced the expression of cell membrane lipid caveolae, caveolin 1. This is intriguing because caveolin 1 influences the endocytic uptake of *E. coli* through caveolae (21). Rho proteins can often be regulated by localizing to caveolae and interacting with caveolins (22). Interestingly, in contrast to our caveolin-1 results, in post-infection LD4-PP-treated cells, we observed an increased expression of Rho GTPase B. This observation is of particular notion as a lower expression of RhoB in human uroepithelial cells is known to increase the intracellular bacterial load (27). Our data suggests that the LD4-PP-mediated alteration in cellular membrane could reflect the lower rate of infection in human uroepithelial cells.

Upon bacterial infection, human uroepithelial cells are known to trigger free radicals (28). LD4-PP differentially regulated the expression of NO in the human uroepithelial cells. *NOS2* mRNA was found to be upregulated, whereas at the protein level, this was inversely proportional to the bacterial load. This discrepancy may be explained by the infection and the well-known IL-1β-mediated regulation of *NOS2* expression via ERK1/2 and STAT1α signaling pathways (29).

Virulence factors from uropathogenic *E. coli* are known to activate NLRP3- and caspase 1-mediated inflammasome in bladder epithelial cells (30) (31). LD4-PP was found to trigger NLRP3 without altering ASC mRNA. However, caspase 1 mRNA was downregulated in LD4-PP-treated cells. This alteration in the inflammasome pathway could highlight a differential priming signaling leading to the expression of the cytokine IL-1β without involving the activation signaling pathway (32). This observation is further supported by IL-1β where *E. coli*-infected and LD4-PP-treated cells showed a lower IL-1β expression compared to *E. coli*-infected cells alone. This indicates that while both LD4-PP and *E. coli* infection trigger IL-1β release, treatment with LD4-PP is able to alleviate the overstimulation of uroepithelial cells.

Apart from the inflammasome pathway, uropathogenic *E. coli* is known to induce a series of extrinsic and intrinsic cascades to initiate uroepithelial apoptosis (33). Several reasons, such as differentially induced uroplakin III expression, promote uroepithelial cell death upon response to uropathogenic *E. coli* (34), and ferritinophagy helps in the bacterial persistence in uroepithelial cells (35). We therefore investigated the effect of LD4-PP on host cell death. However, in the concentrations used, caspase 3 and caspase 9 revealed non-cytotoxic effects and a strong host protective effect of LD4-PP against *E. coli*-infected cells. This host-beneficial effect is relevant as *E. coli* is known to induce nuclear damage in infected cells (36). Furthermore, our data were supported with healthy mitochondria and lysosomes in LD4-PP-treated *E. coli*-infected cells. Our data show that LD4-PP itself does not induce epithelial cell death, while uropathogenic *E. coli* (CFT073) causes pronounced apoptosis that is rescued by LD4-PP. This was evident from reduced caspase 3 and caspase 9 expression and preserved mitochondrial and lysosomal integrity (**Figure 7**) (33, 34, 37).

## Conclusion

Overall, this work highlights the potential of a novel synthetic AMP as a possible therapeutic agent to prevent and treat UTIs. The peptide LD4-PP shows strong antibacterial activity against uropathogens, particularly against drug-resistant bacteria. Furthermore, the host-protective effect proves that this peptide is a strong candidate for future clinical development and application.

## Data Availability

The original contributions presented in the study are included in the article/**Supplementary Material**. Further inquiries can be directed to the corresponding author.
