# Supplementary material for: Synthetic antimicrobial peptide LD4-PP protects the host against E. coli-induced cell death
Source: Front Immunol. 2025 Dec 3;16:1705805. doi: 10.3389/fimmu.2025.1705805 (PMC12708336; doi:10.3389/fimmu.2025.1705805)
Supplement: Supplementary file 1 [file Table1.docx]

***Supplementary file***

**Synthetic antimicrobial peptide LD4-PP protects the host against *E. coli* induced cell death**

Soumitra Mohanty ^1, 2, 3, $, #^, John Kerr White ^1, 2 $^, Yundi Yin ^1, 2$$^ , Taj Muhammad ^3$$^, Isak Demirel ^4^, Adam A. Strömstedt ^3^, Sunithi Gunasekera ^3^, Natalia Ferraz ^5^ Ulf Göransson ^3^, Annelie Brauner ^1, 2*^

^1^ Department of Microbiology, Tumor and Cell Biology, Karolinska Institutet, 171 65 Stockholm, Sweden

^2^ Division of Clinical Microbiology, Karolinska University Hospital, Stockholm, Sweden

^3^ Pharmacognosy, Department of Pharmaceutical Biosciences, Biomedical Centre, Uppsala University, Box 591, 751 24 Uppsala, Sweden

^4^ School of Medical Sciences, Örebro University, Örebro, Sweden.

^5^ Nanotechnology and Functional Materials, Department of Materials Science and Engineering, Uppsala University, Box 35, 751 03 Uppsala, Sweden

*****Corresponding author: Annelie Brauner, Division of Clinical Microbiology, Department of Microbiology, Tumor and Cell Biology, Karolinska Institutet and Karolinska University Hospital, 17165 Stockholm, Sweden

^$^ Equal contribution

^$$^ Equal contribution

^#^ Present address: Biotechnology Research Innovation Council-National Institute of Biomedical Genomics (BRIC-NIBMG), Kalyani 741251, India

**Table 1:** List of primers and probes used in this study.

| **Gene name** | **Sequence (5’-3’)** |
| --- | --- |
| Human *CAV1* (Forward) | AGA CGA GCT GAG CGA GAA GC |
| Human *CAV1* (Reverse) | TCG ATC TCC TTG GTG TGC G |
| Human *RHOB* (Forward) | CAT TCT GAC CAC ACT TGT ACG C |
| Human *RHOB* (Reverse) | GGT TTC TTT TCC CTC TCC TTG T |
| Human *NOS2* (Probe) | Hs01075529_m1 |
| Human *NRF2* (Forward) | TCT GAC TCC GGC ATT TCA CT |
| Human *NRF2* (Reverse) | GGC ACT GTC TAG CTC TTC CA |
| Human *KEAP1* (Forward) | TTC GCC TAC ACG GCC TC |
| Human *KEAP1* (Reverse) | GAA GTT GGC GAT GCC GAT G |
| Human *HMOX1* (Forward) | CTT TTC AGA AGG GCC AGG TGA |
| Human *HMOX1* (Reverse) | GTA GAC AGG GGC GAA GAC TG |
| Human *NLRP3* (Forward) | TGA AGA AAG ATT ACC GTA AGA AGT ACA GA |
| Human *NLRP3* (Reverse) | GCG TTT GTT GAG GCT CAC ACT |
| Human *ASC* (Forward) | CGC GAG GGT CAC AAA CGT |
| Human *ASC* (Reverse) | TGC TCA TCC GTC AGG ACC TT |
| Human *CASPASE1* (Forward) | TCC CTA GAA GAA GCT CAA AGG ATA TG |
| Human *CASPASE1* (Reverse) | CGT GTG CGG CTT GAC TTG |
| Human *ITGB1* (Probe | Hs00559595_m1 |
| Human *UPK1A* (Probe) | Hs00199638_m1 |
| Human *IL1B* (Forward) | CAC GAT GCA CCT GTA CGA TCA |
| Human *IL1B* (Reverse) | GTT GCT CCA TAT CCT GTC CCT |
| Human *IL6* (Forward) | TTC GGT CCA GTT GCC TCT C |
| Human *IL6* (Reverse) | TGG CAT TTG TGG TTG GGT CA |
| Human *CXCL8* (Forward) | AAG AGA GCT CTG TCT GGA CC |
| Human  *CXCL8* (Reverse) | GAT ATT CTC TTG GCC CTT GG |
| Human *ACT B* (Forward) | AAG AGA GGC ATC CTC ACC CT |
| Human *ACT B* (Reverse) | TAC ATC GCT GGG GTG TTG |

**Table 2:** List of antibodies used in this study.

| **Protein name** | **Primary antibody** | **Primary antibody dilution** | **Secondary**  **antibody** | **Secondary**  **antibody dilution** |
| --- | --- | --- | --- | --- |
| Caveolin 1 | Santacruz | 1:200 (IF) | Alexa 647, Invitrogen | 1:400 (IF) |
| RhoB | Invitrogen | 1:200 (IF) | Alexa 488, Invitrogen | 1:400 (IF) |
| NOS2 | Santacruz | 1:100 (IF) | Alexa 594, Invitrogen | 1:400 (IF) |
| Mitosox | Invitrogen | N/A | N/A | N/A |
| Phalloidin | Invitrogen | 1:1000 (IF) | N/A | N/A |
| Caspase 3 | Invitrogen | 1:200 (IF) | Alexa 488, Invitrogen | 1:400 (IF) |
| Caspase 9 | Santacruz | 1:200 (IF) | Alexa 488, Invitrogen | 1:400 (IF) |
| Lysosome | Invitrogen | 1:200 (IF) | Alexa 647, Invitrogen | 1:400 (IF) |
| Mitochondria | Invitrogen | 1:200 (IF) | Alexa 594, Invitrogen | 1:400 (IF) |

**Supplementary Figure S1.** Viability of uroepithelial cells treated with LD4-PP


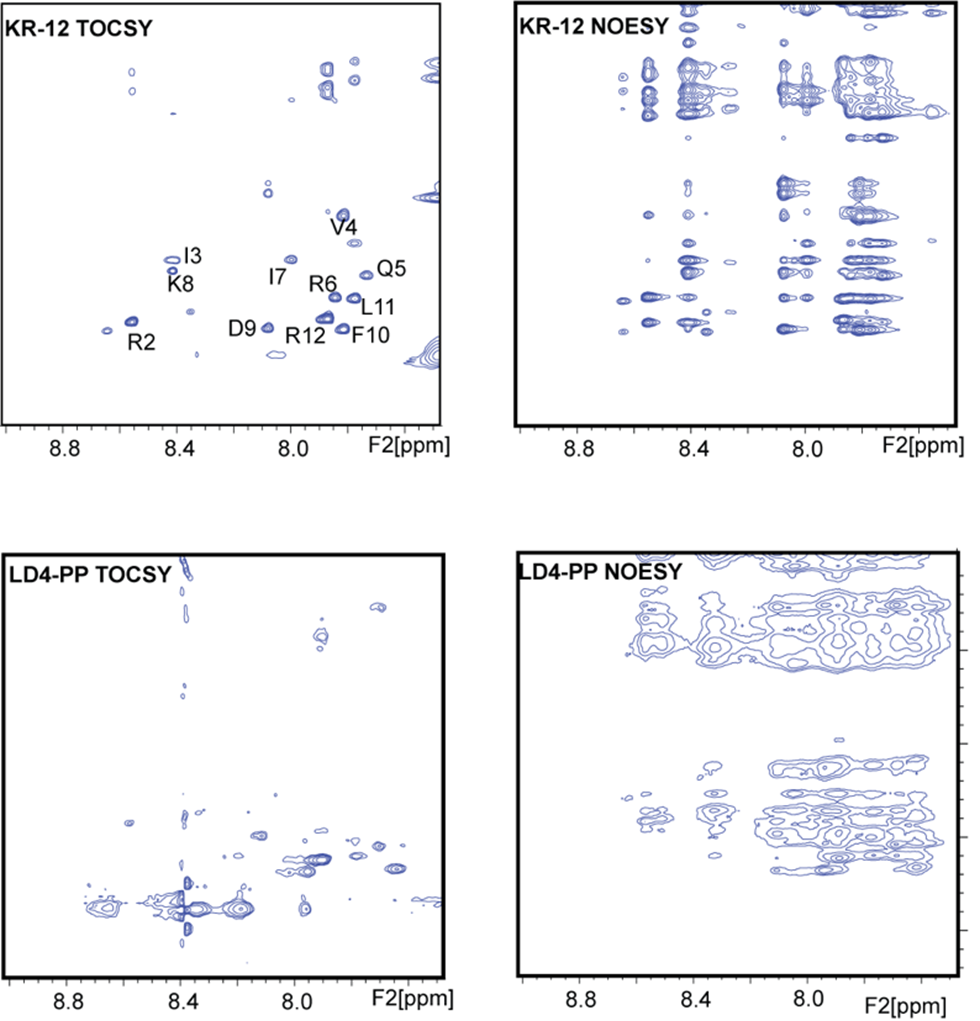


**Supplementary Figure S2. KR-12 and LD4-PP 2D TOCSY and NOESY spectra in the presence of SDS-micelles.** The 2D TOCSY and NOESY spectra of KR-12 in SDS-micelles exhibit well-dispersed peaks, enabling resonance assignments. Conversely, 2D spectra of LD4-PP show a broadening of signals and overlapping resonances, hindering accurate-specific chemical shift assignments.

**
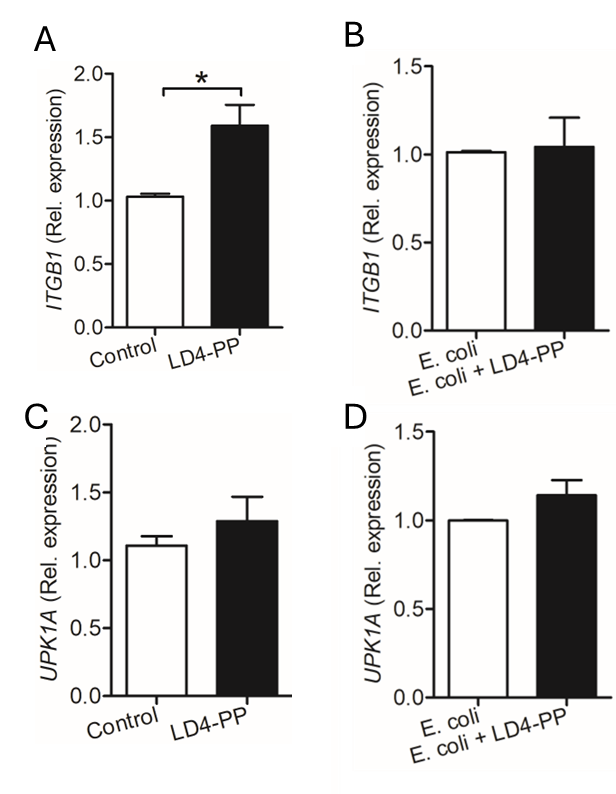
**

**Supplementary Figure S3. LD4-PP differentially regulates cell surface receptors.** Expression of *ITGB1* mRNA **(A)** upon 5µM LD4-PP treatment, **(B)** 1h *E. coli* infection followed by 1h of 5µM LD4-PP treatment in human uroepithelial cells, 5637 (n=4). Likewise, expression of *UPK1A* mRNA **(C)** upon 5µM LD4-PP treatment, **(D)** 1h infection followed by 1h of 5µM LD4-PP treatment in human uroepithelial cells, 5637 (n=4). Data are shown as mean + SEM. Significance levels mentioned as *p ≤ 0.05.


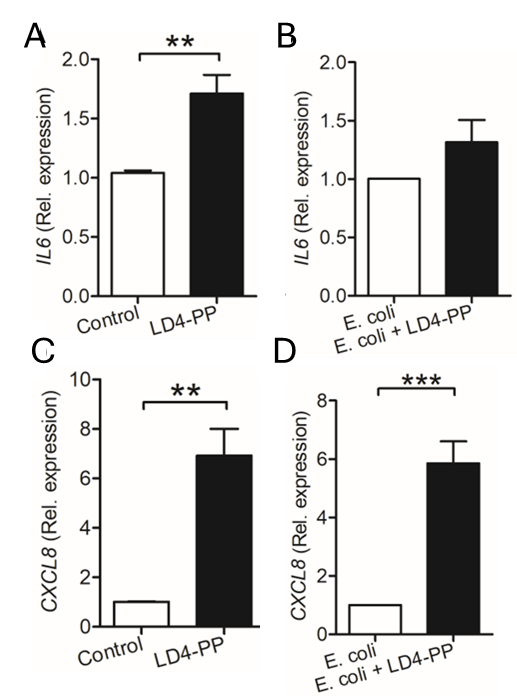


**Supplementary Figure S4. LD4-PP regulates cytokine expression.** Expression of *IL6* mRNA **(A)** upon 5µM LD4-PP treatment, **(B)** 1h *E. coli* infection followed by 1h of 5µM LD4-PP treatment in human uroepithelial cells, 5637 (n=4). Similarly, expression of *CXCL-8* mRNA **(C)** upon 5µM LD4-PP treatment, **(D)** 1h infection followed by 1h of 5µM LD4-PP treatment in human uroepithelial cells, 5637 (n=4). Data are shown as mean + SEM. Significance levels mentioned as **p ≤ 0.01 and ***p ≤ 0.001.
